# Holistic Management of Multiple Myeloma: A Case Report on Siddha Therapy as an Adjunct to the Bortezomib-Lenalidomide-Dexamethasone (BLD) Regimen

**DOI:** 10.7759/cureus.84203

**Published:** 2025-05-15

**Authors:** Bharathi Baskar, Akila B

**Affiliations:** 1 Central Council for Research in Siddha, Siddha Clinical Research Unit, Safdarjung Hospital, New Delhi, IND

**Keywords:** herbal, herbo-mineral, integrative treatment, multiple myeloma, siddha medicine

## Abstract

Multiple myeloma (MM) is a complex haematological malignancy characterized by the clonal proliferation of plasma cells within the bone marrow. It is the second most common haematological malignancy that presents in elderly patients. The integration of complementary medicine into conventional cancer care is increasingly observed among patients, driven by factors such as symptom management, holistic well-being, and cultural alignment. This case report presents the integrated management of a 65-year-old male patient diagnosed with MM, who received both chemotherapy and Siddha medicine to alleviate constitutional symptoms and improve overall well-being. The patient complained of weakness, easy fatigability, and insomnia. The diagnosis of MM was established elsewhere through serum protein electrophoresis, immunofixation, and bone marrow biopsy. He was treated with the bortezomib, lenalidomide, and dexamethasone (BLD) regimen along with a combination of Siddha herbal and herbo-mineral formulations. After six months, the patient demonstrated significant improvements in haematological parameters, general physical condition, and quality of life. This case highlights the potential benefits of integrating traditional Siddha medicine with conventional allopathic therapy in managing MM.

## Introduction

Multiple myeloma (MM) is a malignant proliferation of plasma cells within the bone marrow, leading to complications such as bone pain, anaemia, renal dysfunction, and hypercalcemia. It accounts for approximately 1% of all cancers and 10% of haematologic malignancies globally, with an age-standardized incidence of 0.54 to 5.3 cases per 100,000 individuals [1]. The median age at diagnosis is 69 years. Despite therapeutic advances including proteasome inhibitors, immunomodulatory drugs, and autologous stem-cell transplantation, MM remains incurable, with most patients experiencing multiple relapses [2]. The current standard of care includes combination regimens such as bortezomib, lenalidomide, and dexamethasone (BLD), which have improved progression-free survival compared to high-dose chemotherapy alone [3]. However, these regimens are associated with substantial toxicities, including neutropenia, thrombocytopenia, anaemia, peripheral neuropathy, and gastrointestinal disturbances. In addition to physical side effects, patients often report psychological distress, insomnia, fatigue, and reduced appetite, factors that collectively reduce quality of life during treatment. These limitations have driven interest in integrative oncology, an emerging field that combines evidence-based complementary therapies with conventional cancer treatments to manage side effects, enhance patient well-being, and potentially improve therapeutic outcomes. Surveys suggest that 40-84% of cancer patients use some form of complementary medicine, particularly to address stress, fatigue, and treatment-related symptoms [4]. Emerging evidence from cancers such as pancreatic adenocarcinoma suggests that certain complementary approaches, including traditional medicines and dietary interventions, may act synergistically with chemotherapy by modulating resistance pathways and improving tolerability [5]. This raises the question of whether similar strategies could be beneficial in MM.

Siddha medicine, one of India's oldest traditional systems, offers a distinctive framework for integrative care. It is based on the theory of three fundamental bio-energies or doshas: Vatham (movement and neurological function), Pitham (metabolism and energy), and Kapham (structure and lubrication), whose imbalance is believed to cause disease. Siddha treatments typically involve herbal, mineral, and metal-based formulations, as well as dietary and lifestyle modifications aimed at restoring doshic equilibrium and supporting systemic resilience. Siddha formulations often combine herbs with detoxified metals/minerals and exhibit multimodal pharmacodynamic effects, including immunomodulation, apoptosis induction, and oxidative stress reduction. While their pharmacokinetics (absorption, bioavailability) remain understudied, traditional preparation methods aim to enhance therapeutic efficacy and safety.

This case report presents the adjunctive use of Siddha medicine alongside the BLD regimen in a patient with MM. We explore its potential to reduce symptom burden, improve quality of life, and support treatment tolerability. By bridging traditional principles and modern haematologic oncology, this report seeks to contribute to the growing body of evidence on integrative approaches in cancer care.

## Case presentation

Patient history

A 65-year-old male patient visited the Siddha Clinical Research Unit Outpatient Department, Safdarjung Hospital, New Delhi, with complaints of weakness, easy fatigability, facial puffiness, constipation, and insomnia persisting for one and a half years. The symptoms were gradual in onset, evolving over a year, beginning with progressive fatigue and constipation, followed by facial puffiness and disturbed sleep. He also experienced loss of appetite and weight loss (approximately 5 kg over six months). He had a past medical history of pneumonia, which required hospitalization one year prior. During the diagnostic workup for pneumonia, he was incidentally found to have pancytopenia.

Clinical examination

On examination, the patient appeared pale and fatigued. He exhibited facial puffiness. His vital signs were stable, with no fever or hypertension. The patient's pulse was assessed as Kapha-Vatham dominance. In Siddha medicine, Naadi (pulse) diagnosis is a traditional technique used to assess the dominant dosha by palpating the radial pulse with three fingers. It evaluates qualitative features like pulse strength and rhythm to identify physiological imbalances. In this case, Kapha-Vatham dominance was identified. Increased Kapham is associated with indigestion, lethargy, fatigue, pallor, and heaviness of the body, while increased Vatham is linked to wasting, constipation, insomnia, and pain.

Diagnostic findings

**Figure 1:**
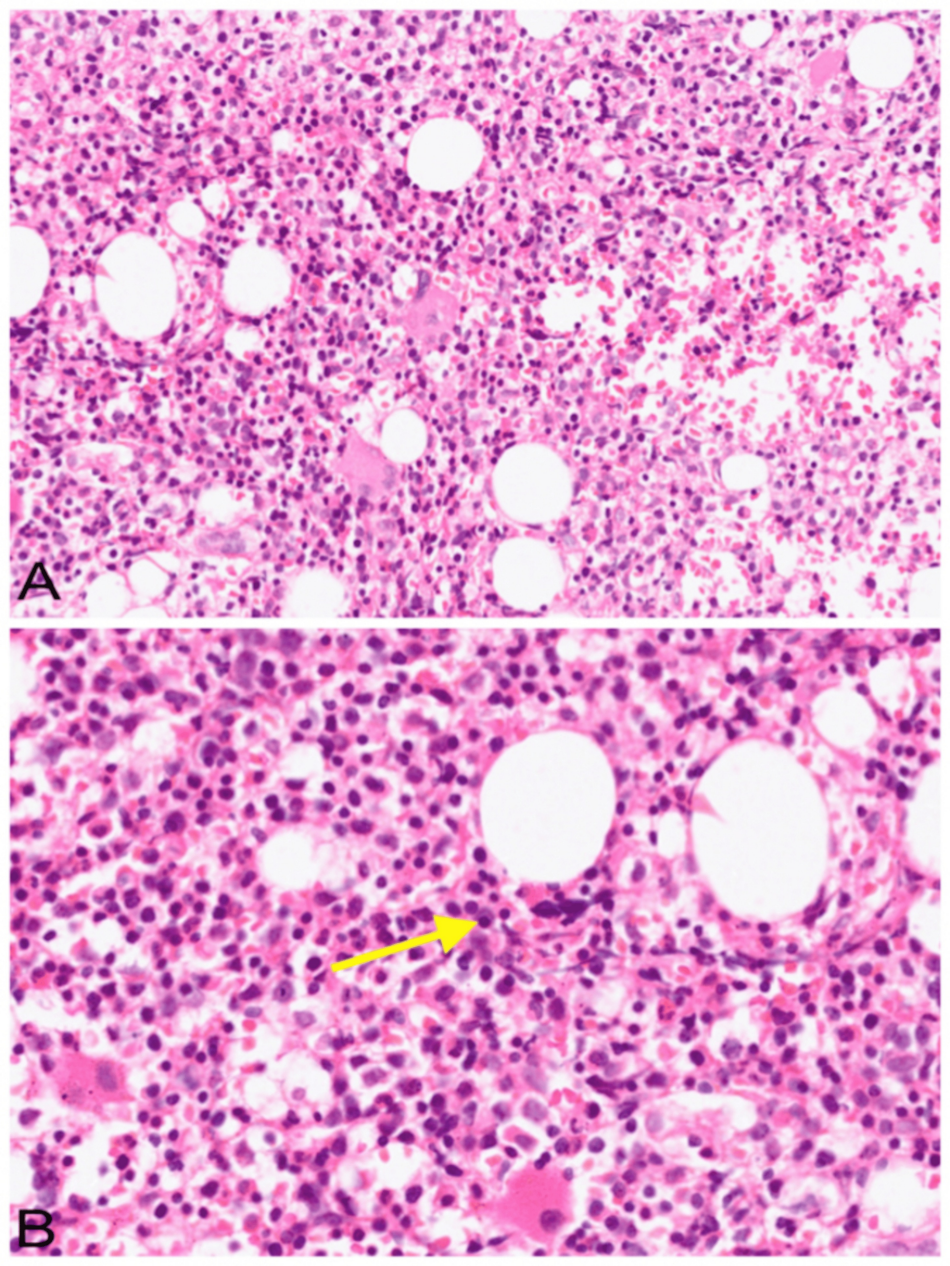
(A) Bone marrow biopsy showing an interstitial increase in plasma cells (haematoxylin and eosin stain 200×); (B) higher magnification showing many plasma cells (yellow arrow) with eccentric nuclei and a perinuclear halo (haematoxylin and eosin stain, 400×)

**Figure 2:**
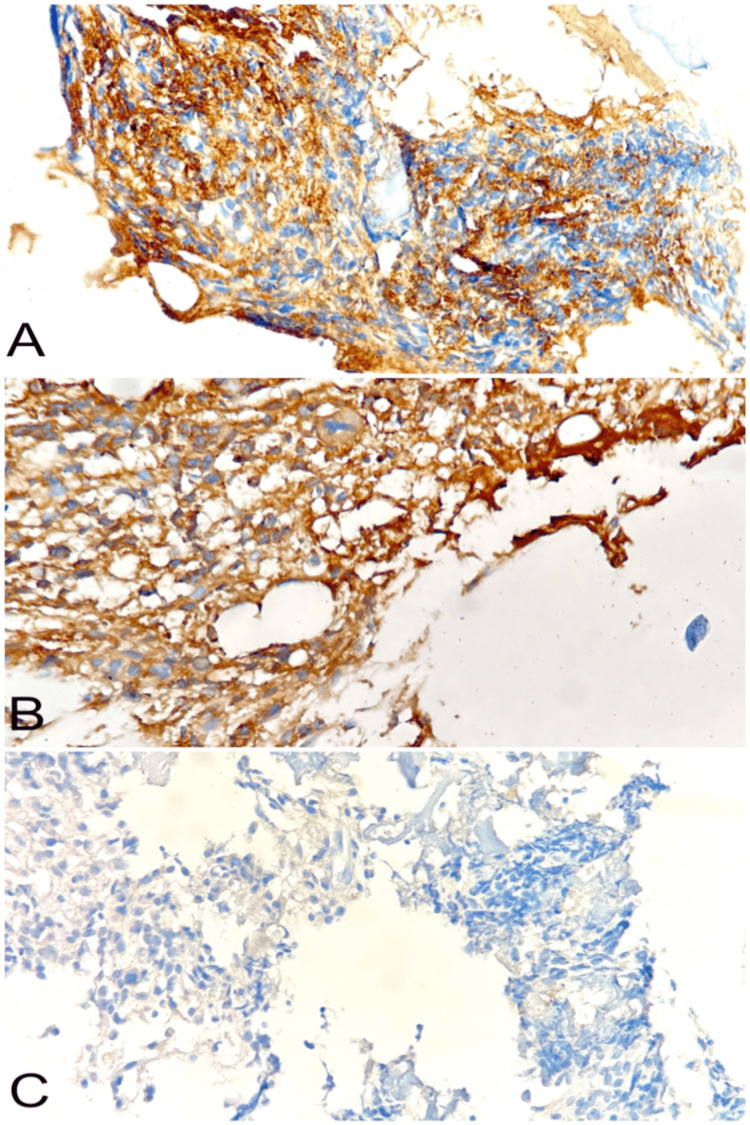
(A) CD138 immunostaining positivity in the plasma cells; (B) the kappa light chain is diffusely positive in the plasma cells; (C) the lambda light chain is negative, indicating clonality restriction to kappa

During the visit, his haemoglobin was low, indicating severe anaemia with leukopenia and thrombocytopenia. Peripheral smear showed Rouleaux formation. Bone marrow biopsy showed an interstitial increase in plasma cells (30%), including binucleate and immature forms (Figure 1). These plasma cells were highlighted by the CD138 immunostain (Figure 2A) with a restriction to the kappa light chain (Figure 2B) while negative for the lambda light chain (Figure 2C), confirming the clonal nature of the plasma cells. Fluorescent in-situ hybridization studies detected FGFR3:IgH rearrangement in 57% of cells, a genetic abnormality associated with aggressive disease and poor prognosis. Serum protein electrophoresis and immunofixation showed an M band and increased serum-free kappa chain. Refer to Table 1 for the detailed values. A diagnosis of MM was made based on the investigations. The Revised Multiple Myeloma International Staging System (R-ISS) was Stage III. All diagnostic evaluations were conducted at the same institute where he received BLD therapy.

**Table 1:** Comparative blood parameters before and after treatment OPD: outpatient department; WBC: white blood cells

Blood parameters	Baseline value (at the time of Siddha OPD visit)	Post-treatment value (after 6 months)	Normal range
Haemoglobin (g/dL)	7	11.1	13–17 (males)
WBC (×10^9^/dL)	3.15	8.69	4.0–11.0
Platelets (×10^9^/dL)	35	89	150–450
Serum-free kappa light chain (mg/L)	157.75	28.32	3.3–19.4
Serum-free kappa/lambda ratio	30.75	20.05	0.26–1.65
M band (g/dL)	5.7 (62.5%)	0.2 (5.6%)	Absent (normal)
Beta 2 microglobulin (mg/L)	5.07	-	0.8–2.1
Urea (mg/dL)	24	27	15–46
Creatinine (mg/dL)	1.0	0.9	0.66–1.25
Albumin (g/dL)	3.9	4.3	3.5–5

Treatment protocol

The patient visited the Siddha clinic during the end of his third cycle of the BLD triplet induction regimen: bortezomib (1.3 mg/m^2^ subcutaneous on days 1, 8, and 15 weekly), lenalidomide 25 mg daily (on days 1-14), and dexamethasone 20 mg (on days 1, 8, and 15) in a nearby tertiary care hospital with a plan of haematopoietic stem-cell transplant. There was persistent fatigue, insomnia, and constipation despite initial haematological stabilization. Following the discussion of risks/benefits, the patient opted to continue conventional chemotherapy alongside Siddha treatment under supervision. This was a patient-centred, informed decision. Siddha formulations were prescribed to improve blood parameters and counteract the malignancy (Table 2). The total duration of the Siddha medication was six months, with a six-week follow-up for monitoring of side effects like nausea, vomiting, and gastritis. During the treatment period, his renal and liver function tests were monitored, which remained unaltered. There were no herbal drug interactions. The patient did not receive any blood transfusions for anaemia during this period.

**Table 2:** Siddha medicine protocol with dosage and mechanism of action

S. No.	Drug Siddha Name	Drug English Name	Dosage	Adjuvant	Mechanism
1.	Capsule Rasagandhi Mezhugu	Herbo-mineral formulation	500 mg BD for 6 months	Palm jaggery	Anti-cancer [6,7]
2.	Tablet Amukkara Chooranam	*Withania somnifera* (Ashwagandha) powder tablet	2 × BD for 6 months	Warm water	Antioxidant, anti-inflammatory, to counteract insomnia [8]
3.	Ayabringaraja Karpam	Elemental iron, ferric oxide, juice of *Eclipta prostrata*, and citrus lemon	200 mg BD for 6 months	Honey	Haematinic, antimicrobial [9]
4.	Syrup Nellikai Legiyum	Indian gooseberry (*Emblica officinalis*) syrup	5 g BD for 6 months	Warm water	Antioxidant, anti-cancer [10]
5.	Syrup Madhulai Manapagu	Pomegranate (*Punica granatum*) herbal syrup	5 mL BD for 6 months	Warm water	Antioxidant, haematinic [11,12]
6.	Nilavembu Kudineer	*Andrographis paniculata* decoction	30 mL HS 3 times a week for 6 months	-	Antipyretic, anti-inflammatory, analgesic, antioxidant [13]
7.	Tablet Bhavana Kadukkai	Purified *Terminalia chebula* (black myrobalan) tablet	2 × BD for 6 months	-	Anti-proliferative, anti-inflammatory, antioxidant [14]

After six months of Siddha treatment in addition to the allopathy therapy regimen, the patient showed improvements in both clinical symptoms and blood parameters, which can be seen in Table 1. Based on the International Myeloma Working Group (IMWG) response criteria, the patient achieved a very good partial response (VGPR) following three cycles of combined BLD chemotherapy and Siddha therapy, with a >90% reduction in serum M-protein and significant improvements in haemoglobin, white cell count, and platelet levels. He reported reduced fatigue, improved appetite, better sleep, and resolution of facial puffiness. The patient's general health and quality of life improved significantly.

## Discussion

MM is characterized by clonal plasma cell proliferation in the bone marrow, leading to a triad of characteristic clinical manifestations: (1) haematologic complications including anaemia (causing fatigue, weakness, and pallor) and immunodeficiency with recurrent infections; (2) skeletal involvement with osteolytic lesions, bone pain, and pathologic fractures; and (3) renal dysfunction due to free light chain deposition. This patient presented with symptoms due to anaemia and had a past history of pneumonia requiring hospitalization. While modern therapies have improved outcomes, disease progression remains driven by complex molecular mechanisms contributing to therapeutic resistance [15,16].

Integrative medicine treatment is classified into four types by the National Center for Complementary and Integrative Health (NCCIH). These approaches include (1) nutritional strategies (e.g., anti-inflammatory diets, vitamin D supplementation, and herbal adjuvants like curcumin); (2) psychological interventions (e.g., mindfulness-based stress reduction for treatment-related anxiety); (3) physical modalities (e.g., acupuncture for chemotherapy-induced nausea or yoga for fatigue); and (4) multimodal programs integrating psychosocial and physical care (e.g., cognitive behavioural therapy). They address chronic stress, poor sleep, sedentary behaviour, and vitamin D deficiency, as these factors influence treatment tolerance, recurrence risk, and survival [17]. A European survey found that 35.9% of cancer patients use complementary therapies, underscoring the need for clinician-guided integration to ensure safety and synergy with conventional treatments [18].

To date, there are no published clinical reports of Siddha-BLD combination therapy in MM. Based on the IMWG response criteria, the patient achieved a VGPR following three cycles of combined BLD chemotherapy and Siddha therapy. These haematologic changes are in line with standard outcomes reported for BLD therapy alone, which typically yields VGPR or better in 40-60% of patients after four to six cycles. However, the patient also reported marked improvements in fatigue, sleep quality, and bowel regularity shortly after the initiation of Siddha therapy, symptoms that often persist despite chemotherapy. While this is a single case, and causality cannot be established, the combination may reflect a supportive or synergistic effect, particularly in quality-of-life domains, warranting further investigation in controlled settings.

The principle of Siddha treatment for MM will be the restoration of dosha imbalance, i.e., excess of Kapham and Vadham. In Siddha literature, MM correlates with "Iratta Vippuruti," which translates to abnormal proliferation of cellular components of blood. The Siddha treatment protocol includes a combination of herbal and herbo-mineral formulations with anti-cancer, anti-inflammatory, antioxidant, and haematinic properties. Capsule Rasagendhi Mezhugu demonstrated anti-cancer activity by inducing apoptosis and inhibiting cancer cell proliferation in various studies [6,7]. This activity has been performed on cervical cancer and prostate cancer cell line studies, which are solid tumours and not on human clinical studies. The mechanism of action of this drug is apoptosis induction via caspase activation and inhibition of NF-κB-mediated proliferation. Interestingly, these are the pathways implicated in the pathogenesis and therapeutic resistance of MM [15]. Though direct evidence in MM models is lacking, these shared molecular targets suggest plausible biological relevance. Nellikai Legiyam is rich in vitamin C and iron, which boosts immunity and exhibits cytotoxic, antioxidant, and antiproliferative effects [19]. Madhulai Manapagu contains pomegranate-derived antioxidants (e.g., phenolic acids, tannins, flavonoids) that exhibit anti-inflammatory, anti-tumorigenic, and chemopreventive properties [20]. Ayabringaraja Karpam has hematinic activity and improves overall vitality [9]. Nilavembu Kudineer and Bhavana Kadukkai have anti-inflammatory, analgesic, and antioxidant effects, helping with pain and fatigue, which has been demonstrated in animal studies [13,14]. The BLD regimen targets clonal plasma cells, while Siddha formulations address the constitutional symptoms. This synergistic approach improves patient adherence to therapy.

Limitation

Without a comparator, it is difficult to isolate the effect of Siddha therapy on the patient's improved parameter. Despite receiving the standard of care, the patient visited our clinic to improve his persisting complaints, which also overlap with the disease process. We wish to present and highlight this integrative approach as an opportunity for further clinical trials and do not claim that the improvement is due to Siddha treatment alone. We strongly advocate for all patients to continue with the standard of care and take complementary treatment after understanding the risks/benefits. Informed consent was obtained prior to the initiation of Siddha treatment and for the publication of the manuscript.

## Conclusions

This case report explores the potential of integrating Siddha medicine with the BLD regimen in a patient with MM, observing improvements in symptom burden, haematological parameters, and quality of life. While these outcomes align with the expected effects of standard therapy, the adjunctive role of Siddha interventions, particularly in mitigating chemotherapy-related toxicities and enhancing patient resilience, warrants further investigation. Given the inherent limitations of a single-case design (e.g., lack of comparator group, confounding variables), the observed benefits cannot be definitively attributed to Siddha therapy. However, the absence of adverse interactions and the patient's holistic progress suggest that such integrative approaches may merit evaluation in larger, controlled studies. Standard therapy, including BLD and transplantation, remains the cornerstone of MM management; complementary interventions like Siddha should be studied systematically to assess their value as adjuncts to conventional care.

## References

[1] Ludwig H, Novis Durie S, Meckl A, Hinke A, Durie B (2020). Multiple myeloma incidence and mortality around the globe; interrelations between health access and quality, economic resources, and patient empowerment. Oncologist.

[2] Das S, Juliana N, Yazit NA, Azmani S, Abu IF (2022). Multiple myeloma: challenges encountered and future options for better treatment. Int J Mol Sci.

[3] Attal M, Lauwers-Cances V, Hulin C (2017). Lenalidomide, bortezomib, and dexamethasone with transplantation for myeloma. N Engl J Med.

[4] O'Brien K, Ried K, Binjemain T, Sali A (2022). Integrative approaches to the treatment of cancer. Cancers (Basel).

[5] Jentzsch V, Davis JA, Djamgoz MB (2020). Pancreatic cancer (PDAC): introduction of evidence-based complementary measures into integrative clinical management. Cancers (Basel).

[6] Riyasdeen A, Periasamy VS, Paul P, Alshatwi AA, Akbarsha MA (2012). Chloroform extract of Rasagenthi Mezhugu, a Siddha formulation, as an evidence-based complementary and alternative medicine for HPV-positive cervical cancers. Evid Based Complement Alternat Med.

[7] Ranga RS, Girija R, Nur-e-Alam M (2004). Rasagenthi lehyam (RL) a novel complementary and alternative medicine for prostate cancer. Cancer Chemother Pharmacol.

[8] Rajalakshmi P, Vadivel V, Brindha P (2017). Investigation of in vitro antioxidant and anti-inflammatory activities of selected siddha polyherbal formulations. Indian J Pharm Educ Res.

[9] Arumugam RK, Gunalan G, Firdouse KHH, Arivazhagan A (2023). Pre-clinical studies of Siddha formulations advocated for anaemia: a systematic review and meta-analysis. J Appl Pharm Sci.

[10] Yang CJ, Wang CS, Hung JY (2009). Pyrogallol induces G2-M arrest in human lung cancer cells and inhibits tumor growth in an animal model. Lung Cancer.

[11] Meenakshi Sundaram M, Vanitha A, Logamanian M (2020). Pharmacological evaluation of anti-anaemic activity of Siddha herbal formulation - Madhulai Manappagu. IOSR J Dent Med Sci.

[12] Kim ND, Mehta R, Yu W (2002). Chemopreventive and adjuvant therapeutic potential of pomegranate (Punica granatum) for human breast cancer. Breast Cancer Res Treat.

[13] Anbarasu K, Manisenthil KK, Ramachandran S (2011). Antipyretic, anti-inflammatory and analgesic properties of nilavembu kudineer choornam: a classical preparation used in the treatment of chikungunya fever. Asian Pac J Trop Med.

[14] Bag A, Bhattacharyya SK, Chattopadhyay RR (2013). The development of Terminalia chebula Retz. (Combretaceae) in clinical research. Asian Pac J Trop Biomed.

[15] Heider M, Nickel K, Högner M, Bassermann F (2021). Multiple myeloma: molecular pathogenesis and disease evolution. Oncol Res Treat.

[16] Cowan AJ, Green DJ, Kwok M (2022). Diagnosis and management of multiple myeloma: a review. JAMA.

[17] Gowin K, Muminovic M, Zick SM, Lee RT, Lacchetti C, Mehta A (2024). Integrative therapies in cancer care: an update on the guidelines. Am Soc Clin Oncol Educ Book.

[18] Molassiotis A, Fernández-Ortega P, Pud D (2005). Use of complementary and alternative medicine in cancer patients: a European survey. Ann Oncol.

[19] Gul M, Liu ZW, Iahtisham-Ul-Haq Iahtisham-Ul-Haq (2022). Functional and nutraceutical significance of Amla (Phyllanthus emblica L.): a review. Antioxidants (Basel).

[20] Sharma P, McClees SF, Afaq F (2017). Pomegranate for prevention and treatment of cancer: an update. Molecules.

